# Low back pain and causative movements in pregnancy: a prospective cohort study

**DOI:** 10.1186/s12891-017-1776-x

**Published:** 2017-10-16

**Authors:** Saori Morino, Mika Ishihara, Fumiko Umezaki, Hiroko Hatanaka, Hirotaka Iijima, Mamoru Yamashita, Tomoki Aoyama, Masaki Takahashi

**Affiliations:** 1 School of Science for Open and Environmental Systems, Graduate School of Science and Technology, Keio University, 3-14-1 Hiyoshi, Kohoku-ku, Yokohama, Kanagawa 223-8522 Japan; 2 Japan Society for the Promotion of Science, Tokyo, Japan; 3 Kishokai Medical Corporation, Nagoya, Japan; 4 Department of Physical Therapy, Human Health Sciences, Graduate School of Medicine, Kyoto University, Kyoto, Japan

**Keywords:** Activity, Low back pain, Lumbopelvic pain, Motion, Pregnancy

## Abstract

**Background:**

Low back pain (LBP) during pregnancy might be strongly related to posture and movements of the body, and its management is a clinically important issue. The purpose of this study was to investigate the activities related to LBP during pregnancy.

**Methods:**

Participants included 275 women before 12 weeks of pregnancy. The women were evaluated at 12, 24, 30, and 36 weeks of pregnancy. The intensity of LBP was assessed using the Numerical Rating Scale (NRS). Movements related to LBP were investigated by free descriptive answers. Descriptive statistics were used to compile the movements that pregnant women thought induced LBP at each evaluation. Subsequently, a linear regression analysis was performed to evaluate the degree of association of certain movements with LBP using the data of participants who had LBP. The intensity of LBP (NRS score) was specified as the dependent variable, the movements that were related to pain were specified as the independent variables at the analysis. A significance threshold was set at 0.05.

**Results:**

The final sample used in the analyses was 254, 249, 258, and 245 women at 12, 24, 30, and 36 weeks of pregnancy, respectively. There were 16 kinds of movements that induced LBP and all of them were daily activities rather than special movements that require extra task or effort. As pregnancy progressed, less number of participants attributed pain to a specific movement. At all evaluations, movements, especially sitting up, standing up from a chair, and tossing and turning were thought to be related to LBP. Furthermore, standing up from a chair and tossing and turning were significantly related to LBP throughout the pregnancy. In contrast, lying down and sitting up were significantly related to LBP but the relationship did not continue till late pregnancy.

**Conclusions:**

Daily routine activity is related to LBP during pregnancy. These results suggest that recommendations for pregnant women about basic physical movements, such as ways of standing up that reduce the load on the body might be useful in the management of LBP.

## Background

Lumbopelvic pain (LPP) is a common discomfort experienced by women during pregnancy [1]. LPP is known to lower the quality of life for many women not only during pregnancy but also after pregnancy, up to 11 years after pregnancy [2, 3]. LPP is the general term for low back pain (LBP) and pelvic girdle pain (PGP) that are common symptoms during pregnancy [4]. Meanwhile, PGP was proposed to be a problem distinct from LBP in pregnancy and it is recently said that they should be distinguished because of the differences in the etiology and associated factors such as maternal age and body mass index (BMI) [5, 6]. Between the two, LBP is the most common musculoskeletal complaint during pregnancy [7]. Thus, the factors related to LBP during pregnancy should be identified and, if possible, addressed to allow for a more comfortable pregnancy.

Pregnancy-related LBP often adversely affects the activities of pregnant women. For example, previous research using Disability Rating Index reported that back pain during pregnancy causes restriction of activities such as running [8]. Furthermore, pregnant women with LPP sometimes report significantly less daily mobility and need crutches or wheelchair for assistance [9]. The effect of LBP on activities during pregnancy was reported in other several studies by using validated assessment questionnaires of the disability for LBP in pregnancy or original questionnaires [2, 3, 10, 11]. In these investigations, an activity was selected and pregnant women answered the degree of influence of LBP on the selected motions. Likewise, some studies have focused on the influence of certain movements, such as gait and lifting, on LBP in pregnant women [12, 13]. In these studies, the target motion was selected by the investigators. In other words, the movements that aggravate or induce LBP in pregnant women were not revealed. Therefore, we let the participants describe motions in their own words without asking them leading questions as other studies have done.

The prevalence of LBP is thought to increase as pregnancy progresses, especially in late pregnancy due to the weight gain and a shift in the center of gravity [1, 14]. Meanwhile, ligament laxity due to pregnancy-related hormones, a risk factor for LBP during pregnancy, begins in early pregnancy and, therefore, LBP also begins in early pregnancy [15, 16]. To make matters worse, the LBP of early pregnancy is associated with disability and pain intensity of late pregnancy [17]. Therefore, it is important to assess LBP throughout the period of pregnancy.

The purpose of this study was to investigate the activities related to LBP during pregnancy by using a questionnaire with descriptive answers. Additionally, the degree of association of some motions with LBP during pregnancy was also investigated in women throughout the period of pregnancy.

## Methods

The study was carried out in accordance with the guidelines of the Declaration of Helsinki, and the study protocol was reviewed and approved by the Ethics Committee of the Kyoto University Graduate School of Medicine (approval number: E-2076). Written informed consents were obtained from the participants.

### Study design and participants

Pregnant women who were undergoing a prenatal health checkup in the obstetrics and gynecology clinics at Aichi Prefecture, Japan, between May 2014 and December 2014 were invited to participate in this study. The inclusion criterion was <12 weeks of pregnancy. Women with orthopedic disorders or neurological diseases that affect their activities, regardless of pregnancy, were excluded. Those with high-risk pregnancies were also excluded. This is a part of a longitudinal study that investigated the association between pelvic alignment and LPP during pregnancy. Participants were requested to participate in the study at 12, 24, 30, and 36 weeks of pregnancy. These periods were chosen because that is when regular prenatal checkups are performed and, hence, it would be convenient for them. Personal characteristics (age, height, weight before the pregnancy, and number of previous deliveries) were obtained at the time of recruitment according to self-statements of the participants. Additionally, weight was recorded at each of the periods mentioned. Queries of the participants regarding the questionnaire, such as assessment of LPP, were answered by the measurers (midwives or physiotherapists).

### Assessment of LBP and activity related to LBP

At first visit, the presence of LBP in the 2 years preceding the pregnancy was investigated because it is strongly related to LBP during pregnancy [1]. Additionally, participants were asked about LBP at each visit. Using the Numerical Rating Scale (NRS), the intensity of the worst pain experienced between the time points of confirmation of pregnancy and 12 weeks (first investigation), 12 and 24 weeks, 24 and 30 weeks, and 30 and 36 weeks of pregnancy were assessed [18]. The NRS is an 11-point pain rating scale with the lower and higher endpoints representing the extremes of no pain and worst pain, respectively. NRS is believed to have the same sensitivity as Visual Analog Scale (VAS) and is used in, both, clinical and research fields more than VAS because of its strengths and usability [19, 20]. Although there are various opinions about the cut-off values of NRS for detecting meaningful degree of pain, some studies have defined VAS < 10/100 as no pain [21, 22]. Therefore, NRS >0 was defined as the presence of LBP in this study. Locations of the pain were explained by the measurers using a picture of the human body. Then, we investigated the motion related to LBP with the question “If you feel LBP in a particular motion, please describe the motion that induces LBP.” Thus, if they do not think that any particular motion induces pain, they do not answer this question.

### Statistical analysis

Continuous NRS data of pregnant women with LBP at the four time points were compared by using one-way analysis of variance (ANOVA) with post-hoc testing. Additionally, the data of the participants who were followed up and had LBP during all periods (*N* = 113) were compared by using repeated-measure ANOVA to compare the values longitudinally. We compiled the motions that these pregnant women thought induced LBP at the corresponding weeks of pregnancy in descriptive statistics by using answers from the questionnaire for activities related to LBP. The answers were categorized by using content analysis. Two physiotherapists conducted the process. In addition, we analyzed the differences in the presence of LBP during basic activities of daily living (BADL) using chi-square test with the Bonferroni correction. In this test, the presence of LBP during BADL was expressed as a dummy variable as 0 or 1. The chi-square test was performed 3 times (for 12 vs. 24, 24 vs. 30, and 30 vs. 36 weeks of pregnancy) and the results were considered significant if *p* < 0.017. A linear regression analysis using forced entry method was used to evaluate the degrees of association of the motions with LBP using the data of participants who had LBP. The regression analysis was performed for each of the 4 periods. The intensity of LBP (NRS score) was used as a continuous variable and specified as the dependent variable. Simultaneously, BMI at each periods was used as the independent variable. The presence or absence of LBP in the 2 years before pregnancy was expressed as 1 or 0, respectively, and was also used as the independent variable to take it into account as a risk factor. The pain inducing motion was expressed as a dummy variable, 0 or 1, and specified as the independent variable in the analysis. In this analysis, all of the pain-inducing motions were entered in the same regression model simultaneously at each period. Statistical analyses were performed using SPSS version 23.0 (SPSS, Chicago, IL, USA) with a significance threshold set at 0.05.

## Results

**Fig. 1:**
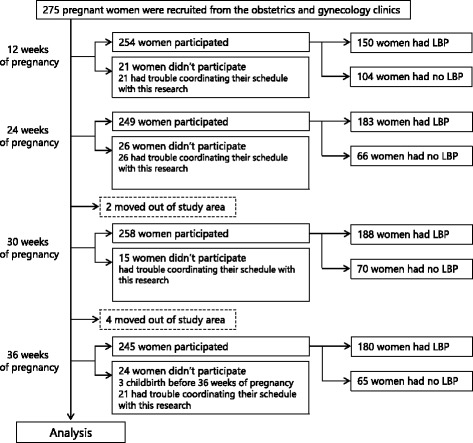
The study sample

Two hundred and seventy-five women who met the inclusion criteria for the survey and agreed to participate in the study were initially enrolled. Among the initially enrolled participants, 21, 26, 17, and 30 women could not participate at 12, 24, 30, and 36 weeks of pregnancy respectively due to discordance between their schedule and the study or delivery before 36 weeks of pregnancy. Therefore, the final sample used in the analyses consisted of the remaining 254, 249, 258, and 245 women at 12, 24, 30, and 36 weeks of pregnancy respectively (Fig. 1). The demographic data of the participants and the prevalence of pain at each period are shown in Table 1. The prevalence of LBP at each of the weeks mentioned was 59.1, 73.5, 72.9, and 73.5%, respectively, and the average intensity of the pain in the participants who had LBP was 4.1 ± 2.3, 4.3 ± 2.1, 4.5 ± 2.3, and 4.8 ± 2.4, respectively (Table 1). The pain intensity at 36 weeks of pregnancy was significantly higher than that at 12 weeks of pregnancy (*p* = 0.045). In the participants who were followed and had LBP during all periods, repeated-measure ANOVA revealed that the pain intensities at 24 (4.8 ± 2.0; *p* = 0.029), 30 (4.7 ± 2.1; *p* = 0.049), and 36 weeks of pregnancy (5.0 ± 2.5; *p* = 0.007) were significantly higher than that at 12 weeks of pregnancy (4.2 ± 2.2). There were 16 kinds of motion that participants thought induced pain (Table 2). The percentage of participants who did not think a specific motion was related to LBP decreased as pregnancy progressed; it was 56.0, 43.2, 47.3, and 31.1% at 12, 24, 30, and 36 weeks of pregnancy. Additionally, the percentage of women who developed LBP during BADL significantly increased between 12 and 24 weeks of pregnancy (*p* = 0.009), and 30 and 36 weeks of pregnancy (p = 0.007) (Table 1). In all investigation periods, the 3 motions that the majority of pregnant women thought were related to LBP were sitting up, standing up from chair, and tossing and turning while supine. The results of linear regression analysis are shown in Table 3. Standing up from a chair was significantly related to LBP at 12 (Regression coefficient and 95% confidence interval: 1.679 [0.37–2.99]), 30 (1.245 [0.21–2.29]), and 36 weeks of pregnancy (1.392 [0.28–2.50]). Tossing and turning was significantly related to LBP at 12 (1.395 [0.02–2.77]), 24 (1.561 [0.52–2.60])), and 36 weeks of pregnancy (1.945 [1.02–2.87]). On the other hand, lying down and sitting up were related to LBP but this relationship did not continue into late pregnancy. Lying down was significantly related to LBP at 24 (1.525 [0.07–3.22]) and 30 weeks of pregnancy (4.799 [0.54–9.06]), and sitting up was significantly related to LBP at 24 weeks of pregnancy (0.936 [0.53–1.82]). In addition, the presence of LBP before pregnancy was related to LBP at 24 weeks of pregnancy (1.274 [0.67–1.88]). R^2^ values for the regression models for the four periods were 0.395, 0.479, 0.331, and 0.466 respectively.

**Table 1:** Demographic characteristics of all participants

	Total	12 weeks of pregnancy	24 weeks of pregnancy	30 weeks of pregnancy	36 weeks of pregnancy
	(*n* = 275)	(*n* = 254)	(*n* = 249)	(*n* = 258)	(*n* = 245)
Age (years)	31.0 ± 4.4				
Height (cm)	158.4 ± 5.6				
Mass (kg)		52.8 ± 8.1	56.9 ± 7.9	59.7 ± 8.1	62.1 ± 7.9
Number of the women having LBP [Prevalence (%)]		150 [59.1]	183 [73.5]	188 [72.9]	180 [73.5]
Average intensity of the pain among the participants who had LBP		4.1 ± 2.3	4.3 ± 2.1	4.5 ± 2.3	4.8 ± 2.4
Median intensity of the pain among the participants who had LBP [25, 75 percentile]		2 [0, 4]	3 [0, 5]	3 [0, 5]	3 [0, 5]
Number of women having LBP during BADL [Percentage (%)]		58 [38.7]	97 [53.0]	95 [50.5]	116 [64.4]

Values are shown as mean ± standard deviation

*LBP* low back pain, *BADL* basic activities of daily living

**Table 2:** Activity related to low back pain

Weeks of pregnancy	Activity	Number of people [Percentage (%)]
		Number of women with LBP = 150
12	Crouching	6 [4.0]
	Lifting heavy objects	2 [1.3]
	Picking child up	4 [2.7]
	Sitting up	15 [10.0]
	Standing up from chair	17 [11.3]
	Tossing and turning	14 [9.3]
	Walking	5 [3.3]
	Others	6 [4.0]
	No answer	84 [56.0]
		Number of women with LBP = 183
24	Crouching	10 [5.5]
	Lying down	6 [3.3]
	Picking child up	3 [1.6]
	Piggyback	2 [1.1]
	Sitting up	26 [14.2]
	Standing up from chair	27 [14.8]
	Tossing and turning	18 [9.8]
	Walking	11 [6.0]
	Washing dishes	2 [1.1]
	Others	4 [2.2]
	No answer	79 [43.2]
		Number of women with LBP = 188
30	Crouching	7 [3.7]
	Lying down	2 [1.1]
	Picking child up	2 [1.1]
	Sitting up	30 [16.0]
	Standing up from chair	27 [14.4]
	Tossing and turning	23 [12.2]
	Walking	9 [4.8]
	Others	4 [2.1]
	No answer	89 [47.3]
		Number of women with LBP = 180
36	Crouching	5 [2.8]
	Driving	4 [2.2]
	Hanging out the washing	2 [1.1]
	Lying down	4 [2.2]
	Picking child up	5 [2.8]
	Piggyback	2 [1.1]
	Sitting up	25 [13.9]
	Standing up from chair	26 [14.4]
	Tossing and turning	37 [20.6]
	Walking	16 [8.9]
	Others	1 [0.6]
	No answer	56 [31.1]

*LBP* low back pain

**Table 3:** Results of multiple regression analysis

12 weeks of pregnancy			24 weeks of pregnancy			30 weeks of pregnancy			36 weeks of pregnancy		
Independent variable	Regression coefficient	95% CI	Independent variable	Regression coefficient	95% CI	Independent variable	Regression coefficient	95% CI	Independent variable	Regression coefficient	95% CI
Crouching	0.929	-1.04-2.90	Cough	2.988	-0.73-6.70	Crouching	0.570	-1.08-2.22	Crouching	0.595	-2.11-3.30
Hanging out the washing	1.251	-1.15-1.71	Crouching	0.571	-0.82-1.96	Hanging out the washing	3.259	-1.01-7.53	Driving	-1.369	-3.80-1.07
Lifting heavy objects	2.639	-0.68-5.96	Hanging out the washing	0.727	-2.98-4.43	Lifting heavy objects	-0.103	-4.37-4.16	Hanging out the washing	0.608	-2.58-3.80
Lying down	0.308	-4.44-5.05	**Lying down**	**1.525**	**0.07-3.22**	**Lying down**	**4.799**	**0.54-9.06**	Lying down	1.469	-1.03-3.96
Picking child up	1.886	-0.40-4.18	Picking child up	-1.600	-4.23-1.03	Picking child up	2.685	-1.95-7.32	Picking child up	-0.105	-2.76-2.55
Piggyback	-2.004	-6.58-2.57	Piggyback	0.958	-1.72-3.64	Piggyback	0.593	-3.71-4.90	Piggyback	-0.611	-5.84-4.62
Sitting up	-0.851	-2.27-0.36	**Sitting up**	**1.102**	**0.05-1.82**	Sitting up	0.376	-0.55-1.30	Sitting up	0.347	-0.66-1.36
**Standing up from chair**	**1.679**	**0.37-2.99**	Standing up from chair	0.486	-0.39-1.36	**Standing up from chair**	**1.245**	**0.21-2.29**	**Standing up from chair**	**1.392**	**0.28-2.50**
Stepping the stairs	-0.234	-4.91-4.44	Stepping the stairs	1.000	-2.72-4.72	Stepping the stairs	-0.944	-5.29-3.40	**Tossing and turning**	**1.945**	**1.02-2.87**
Stretching body	2.011	-2.99-7.01	Stretching body	-0.290	-4.00-3.42	Tossing and turning	0.966	-0.10-2.03	Walking	1.285	-0.08-2.65
**Tossing and turning**	**1.395**	**0.02-2.77**	**Tossing and turning**	**1.561**	**0.52-2.60**	Walking	0.408	-1.38-2.20	Washing dishes	3.523	-3.40-10.45
Vacuum cleaning	4.153	-0.35-8.65	Walking	-0.403	-1.57-0.76	BMI at 30 weeks of pregnancy	0.038	-0.08-0.16	LBP before pregnacy	0.318	-0.44-1.08
Walking	0.174	-2.12-2.47	Washing dishes	0.986	-2.73-4.70	LBP before pregnacy	0.392	-0.29-1.08	BMI at 36 weeks of pregnancy	0.017	-0.12-0.15
BMI at 12 weeks of pregnancy	0.023	-0.11-0.16	BMI at 24 weeks of pregnancy	-0.008	-0.03-4.70						
LBP before pregnacy	0.629	-0.36-1.62	**LBP before pregnacy**	**1.274**	**0.67-1.88**						

The data in bold is statisticaly significant

*CI* confidence interval, *LBP* Low back pain

## Discussion

In this study, certain motions that were related to LBP in pregnant women were investigated by free descriptive answers from early pregnancy to late pregnancy. As the main result, 16 kinds of motions, especially sitting up, standing up from a chair, and tossing and turning, were mentioned by many pregnant women. Additionally, it was revealed that standing up from a chair, tossing and turning, lying down, and sitting up were related to the intensity of LBP significantly.

According to the World Confederation for Physical Therapy, BADL was defined as activities that cover domains such as dressing, eating, mobility, using the toilet, and hygiene [23]. In this study, seven motions such as crouching, lying down, sitting up, standing up from a chair, tossing and turning, and walking were thought to be a part of BADL. Therefore, pregnant women mainly think that daily motions, especially motions that are a part of BADL, are related to LBP. In general, special tasks such as lifting heavy objects and running were identified as the risk factors of LBP during pregnancy [24]. However, Close et al. reported that pregnant women reported that daily activities, such as walking, were disturbed due to LBP in a prospective study using qualitative design [25]. Their results along with those of this study establish that pregnant women have troubles with daily routine activities rather than special motions that require extra task or effort due to LBP. In other words, pregnant women have difficulty in BADL that are essential in daily life because of LBP. BADL includes the fundamental activities of the activities of daily living. The result that BADL is related to LBP during pregnancy indicates that the elementary part in the daily lives of pregnant women might be restricted. Thus, LBP during pregnancy needs to be addressed. Furthermore, the proportion of women who did not think that particular movements were related to LBP increased as their pregnancies progressed. In other words, the assiciation of motions with LBP might be increased in late pregnancy. Moreover, the intensity of pain also had a tendency to increase as previously reported [26]. Thus, the demand for pain management may increase as pregnancy progresses. A pregnancy-specific self-report questionnaire assessing mobility in relation to LPP called the Pregnancy Mobility Index was suggested recently [27]. However, it contains motions that these women seem not to do, such as traveling by bicycle. Thus, the questionnaire might be suitable to assess the effect of LPP on the quality of life; however, the items do not represent the motions that reflect real opinions of pregnant women. That is to say, some of these items might not serve the purpose of asking pregnant women about motions related to LPP because they rarely perform these activities in the first place. On the contrary, the results of this study reflect the opinions of pregnant women and include only the motions that they do perform. Thus, the results provide useful information in understanding what motions might be associated with LBP during pregnancy. The motion that is indispensable in daily life such as BADL, rather than heavy load motions that are thought to be risk factors of LBP, should be investigated for the management of LBP during pregnancy.

In all motions, pregnant women feel LBP especially during sitting up, standing up from a chair, and tossing and turning throughout the pregnancy. Hence, these motions might be strongly correlated with the occurrence of LBP. Furthermore, the previous episodes of LBP before pregnancy and BMI at each periods were considered in the analysis as a risk factor of LBP during pregnancy. There are many factors that may affect LBP during pregnancy and all of them were not considered in this study, unfortunately. However, the result of the analysis after considering the presence of LBP before the pregnancy and BMI is meaningful because these episodes are an important risk factor of LBP [1, 28]. Therefore, it can be thought that these motions have a substantial effect on LBP of the participants after adjustment for LBP before pregnancy and BMI. The motions that were significantly related to LBP according to the results of linear regression analysis, such as standing up from a chair and tossing and turning, are those that commonly need rotation and extension/flexion of the trunk. These trunk motions might be related to LBP throughout the pregnancy. Gilleard et al. reported the change in postural alignment of the thoracolumbar spine in sitting position as pregnancy progresses [29]. Furthermore, changes in the range of motion of the trunk during sitting and standing were observed in pregnancy [30]. Considering these results, the changes in postural alignment during pregnancy might cause changes in movements and cause strain on body segments, which subsequently contribute to musculoskeletal pain during standing up from a chair. Therefore, further studies are needed in order to understand the association between specific motions and LBP during pregnancy. Simultaneously, there is a need for guidelines for these motions, similar to the guidelines regarding occupational weight bearing in pregnancy, to reduce the risk of overexertion disorder [31]. Additionally, the motion of lying down usually includes rotation of the trunk and rotation of the vertebrae. It is believed that axial trunk rotation is related to LBP and improved coordinated trunk movements would be of help in patients with LBP [32, 33]. Furthermore, the trunk needs to be supported while lying down. The stabilizing of the spine itself causes some strain to the soft tissues of the trunk, such as muscles, and lead to LBP [34]. Therefore, lying down might contribute to LBP. In contrast, changes in body position between lying down and standing positions, such as sitting up and lying down, contributed to LBP during only 24 and 30 weeks of pregnancy. A balance between the changes in body weight and gravity begins in this period and pregnant women cannot manage the changes or take large loads on the body during changing of positions [35]. However, the risk factors between particular motions and LBP could not be identified in this study. Simultaneously, R^2^ values of the regression models ranged from 0.331 to 0.479 in this study. These values indicates the regression line did not fit the real data points perfectly. In other words, the results of the analysis could not sufficiently explain the factors related to LBP during pregnancy. One of the reasons for this is that the factors added in the analysis and the number of participants were not enough. Furthermore, it is possible that variables other than those included in the regression model accounted for a large degree of the variance in LBP intensity. Various risk factors are thought to be related to LBP during pregnancy [1, 4], and the study included sufficient information and participants to determine the factors related to LBP during pregnancy is needed. Moreover, there are many other factors that are related to daily motions and involved in LBP. For example, awkward posture by the same posture for a long time affect various daily activities and LBP [36]. Of those various factors, we focused on motions that are related to LBP and can be managed by physical modalities with proper exercise techniques and movement coaching. A more detailed investigation that focuses on characteristics of load on body in these motions, especially taken with regards to other backgrounds, such as daily movements and body condition will make the current results more rewarding.

There were several limitations of this study. First, we investigated the motions related to LBP by free descriptive answers, rather than via validated selection type questionnaire. Thus, the response rate to the question about motion that the participants thought induced LBP was low, between 44.0% and 68.9% at various investigation periods. It can be thought that this is because the remaining participants did not think that any particular motion induces pain and some participants might not remember a particular motion that was related to LBP. Due to this, the possibility that other motions or postures are related to LBP cannot be denied. However, despite the limitation, the opinions of pregnant women on what motions cause LBP were demonstrated without restrictions or preconditions in this study. Second, we did not evaluate other factors that may affect pregnancy-related LBP, such as the level of pregnancy-related hormones, muscular strength, or physical flexibility. Additionally, the detailed mechanism of how particular motions were related to LBP was not revealed. Simultaneously, the longitudinal comparison was difficult in this study because the participants at the four time points were not the same. Owing to these reasons, although we could clarify the main purpose of this study—identify the motions that are strongly related to LBP and need to be investigated in the future—the causal relation between motions and LBP was not revealed. Hence, further research with pregnant women who do not have LBP as controls is required by conducting interventions or observations to evaluate if all pregnant women perform the same movements in the same way, and frequency, and/or by longitudinal study design to investigate the causal relation and other intermediate factors for LBP during pregnancy.

## Conclusions

The results suggest that pregnant women have difficulties in daily routine motion due to LBP rather than special motions that require extra task or effort that are generally thought of as risk factors for LBP. Additionally, standing up from a chair and tossing and turning were significantly related to LBP throughout the pregnancy. In contrast, lying down and sitting up were significantly related to LBP in mid-pregnancy. Therefore, recommendations for pregnant women about basic daily movements such as ways of standing up that reduce the load on the body might be useful in the management of LBP during pregnancy. It is important that the duration of pregnancy and body weight be considered in such recommendations.

## Abbreviations

BADL: Basic activities of daily living
BMI: Body mass index
LBP: Low back pain
LPP: Lumbopelvic pain
NRS: Numerical Rating Scale
PGP: Pelvic girdle pain
